# Demonstration of a Speckle Based Sensing with Pulse-Doppler Radar for Vibration Detection

**DOI:** 10.3390/s18051409

**Published:** 2018-05-03

**Authors:** Nisan Ozana, Reuven Bauer, Koby Ashkenazy, Nissim Sasson, Ariel Schwarz, Amir Shemer, Zeev Zalevsky

**Affiliations:** 1 Faculty of Engineering and the Nano Technology Center, Bar-Ilan University, Ramat-Gan 52900, Israel; bauer@elta.co.il (R.B.); Ariel.Schwarz@biu.ac.il (A.Sc.); shemeramir@gmail.com (A.Sh.); Zeev.Zalevsky@biu.ac.il (Z.Z.); 2 ELTA Systems Ltd., P.O.B. 330 Ashdod 7710202, Israel; kashkenazy@elta.co.il (K.A.); snissim@elta.co.il (N.S.)

**Keywords:** antenna, optical scattering, Pulse-Doppler Radar, receiver, speckle, transmitter

## Abstract

In previous works, an optical technique for extraction and separation of remote static vibrations has been demonstrated. In this paper, we will describe an approach in which RF speckle movement is used to extract remote vibrations of a static target. The use of conventional radar Doppler methods is not suitable for detecting vibrations of static targets. In addition, the speckle method has an important advantage, in that it is able to detect vibrations at far greater distances than what is normally detected in classical optical methods. The experiment described in this paper was done using a motorized vehicle, which engine was turned on and off. The results showed that the system was able to distinguish between the different engine states, and in addition, was able to determine the vibration frequency of the engine. The first step towards real time detection of human vital signs using RF speckle patterns is presented.

## 1. Introduction

The use of optical sensing technology to extract vibration sources has been successfully demonstrated in previous works. In a system described in Z. Zalevsky et al., an optical system was designed and demonstrated for the detection of sounds by projecting a laser beam on a vibrating target and observing the movement of the secondary speckle patterns that are created by the interferences from the target’s roughness [1]. The speckles are self-interference random patterns. It has the unique quality that each individual speckle serves as a reference point. From each speckle point, one may track the changes in the light phase that are being scattered from the target surface. This speckle-pattern interferometry was used for measuring displacements and analyzing vibration frequencies, as well as characteristics of the object deformations. In order to measure the object deformation displacements, the speckle pattern is subtracted before and after the deformation has occurred. The deformations are due to changes in loading, temperature, etc. This procedure produces a speckle pattern that corresponds to the object’s local surface changes between two exposures. From the correlation between the speckle patterns, both the magnitude and the direction of the object’s local surface displacement are able to be determined. 

Other applications which utilize an optical approach for the extraction and separation of remote vibration sources were used for biomedical measurements, such as monitoring heart rate, breathing, blood pressure, blood oximetry, blood coagulation [1,2,3,4], bone fractures [5], melanoma [6], and glucose concentration in blood [7,8]. 

The above applications illuminate the object under observation using a laser beam which produces the secondary speckle pattern. A fast-imaging camera observes the temporal intensity fluctuations of the imaged speckle pattern and its relative movements [9,10,11,12,13]. In this study, we show that the same algorithm used for optical sensing can be used also when the object under measurements is excited by an RF source. Hence, instead of having the image recorded by a camera, the scattering is received and processed using an RF receiver.

In the following experiment, we used RF frequency pulses for generating the secondary speckle patterns. The illuminated object was an automotive vehicle. It was illuminated under two different conditions. The first was when the vehicle engine was turned on, and the second was with the engine turned off.

A series of pulses were transmitted toward the vehicle. The returning pulses were stored in a range bin (discrete data elements received from the reflected radar signal) and processed by applying an image correlation algorithm. We found out that not only was it possible to detect the movement of the vehicle, but it was also possible to determine its vibration frequency.

The use of RF pulses, as opposed to optical pulses, enables us to determine the vibrations of an observed object at far greater distances than what would be possible in an optical system.

In our experiment, a transmitter was utilized and operated at 10 GHz. Increasing the transmitter frequency increases the sensitivity resolution of the entire system. This would enable us to detect vibrations at higher frequencies. However, doing so in an optical system would require using a complicated integration and expensive hardware.

In addition, the use of a Doppler radar enables us to detect both the vibration frequencies and the directional velocity by increasing the sampling rate. As opposed to optics, the radar field-of-view is relatively wide, and can operate without a tracking system as used in optical systems [1].

Several options were considered for illuminating the vehicle. The preferred option for our experiment was using an X-Band, phased array, Pulse-Doppler radar. This radar is normally used for search and detection of personal in the surrounding areas. Its main use is for detection of threats, threat range, and threat velocity. Each radar section has a transmitter (Tx) and receiver (Rx) modules connected directly to the antenna.

The antenna is a phased array antenna consisting of 16 radiating elements. Normally, the 16 antenna beams are combined into a single beam which scans in the horizontal (azimuth) direction. In our demonstration, the scanning option was disabled, and only eight channels were operated simultaneously.

The vertical (elevation) 3 dB beam width was 10°, while the vertical (azimuth) 3 dB beam width was about 100°. The beams were separated by 0.5° and the pulse width was 0.1 ms with a dwell time (illumination time on target) of 100 ms. The tested vehicle was illuminated at ranges varying from 50 m to 120 m.

Note, that even though phased array radar was used in our experiment, many other RF transmitting techniques are also valid. 

## 2. Experimental Setup—Pulse-Doppler Radar

**Figure 1:**
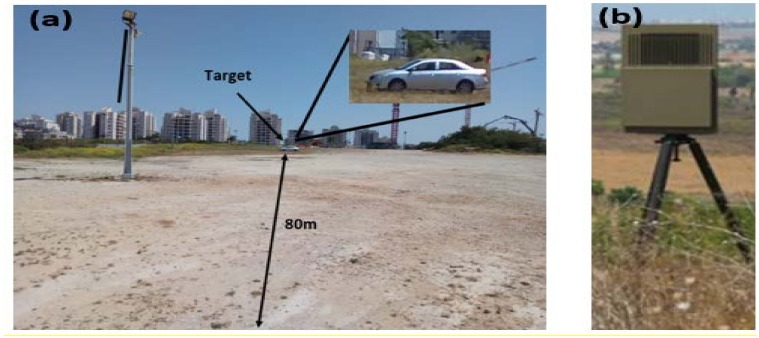
(**a**) View from behind the radar. The target is located 80 m from the radar. The target is shown in the inset; (**b**) ELTA radar used in the experiment.

The setup was consisted of a Pulse-Doppler radar and a vehicle. The measurement was done in an open field at Bar-Ilan University. The radar was placed at one end of the field, while the vehicle (Toyota Corolla Sun, Japan, 2008) was placed at various distances from the radar in front of the radar line-of-sight. The radar system is shown in Figure 1.

The distance between the radar and the vehicle was set to 40, 80, and 120 m, respectively. As was mentioned earlier, the vehicle was illuminated by the radar in two different scenarios. In one scenario, the engine was off, while in the second, the engine was turned on. The measurements of the received pulses were processed using a unique algorithm designed to extract the secondary speckles reflected from the vehicle, and to determine its rate of vibration when the engine was on. Prior to each measurement, a person walked at the targeted site, which was next to the vehicle. This was done in order to provide a reference for detecting the vibration of the vehicle. The discrimination between the movements of a person and a stationary vehicle is possible, since the signal returning from the target was measured using a Pulse-Doppler radar, in which the strength of the received pulse was a function of the target’s movement with respect to the radar.

The principle behind the Pulse-Doppler radar is that it is used to calculate the range to targets by measuring the elapsed time between sending a radio pulse and receiving the reflection from the object [14,15,16,17]. It also utilizes the Doppler effect, where the target’s movement produces a frequency shift on the signal reflected back from the moving target. As the target moves between each transmitted pulse, the returned signal has a phase difference or phase shift from pulse to pulse. Since the movement velocity of a person in reference to the radar is higher than that of the vibrating stationary vehicle, the amplitude of its Doppler frequency will be higher than that of the vehicle, thus enabling it to be used as a relative reference signal.

**Figure 2:**
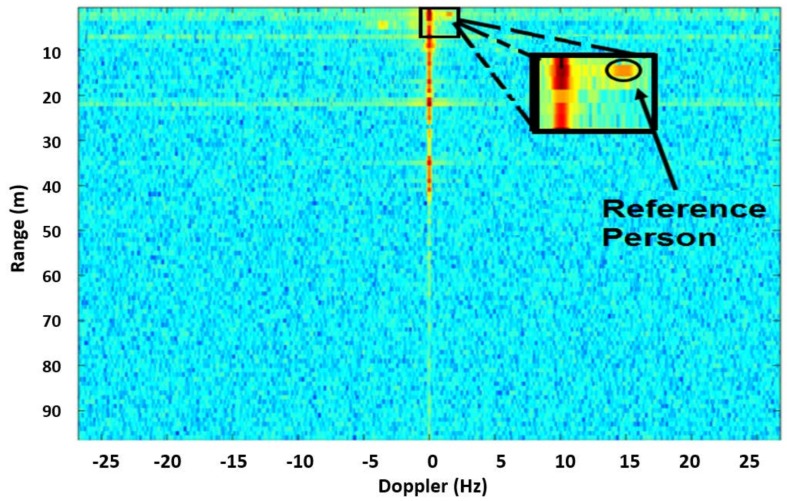
Doppler frequencies vs. range with the vehicle at a distance of 80 m. Each range cell represents 40 m. The target and the reference person are located in range cell 2.

The goal of the pulsed-Doppler signal processing is to extract the desired target signal out of the surrounding noise or the clutter (unwanted echoes). It is done by using techniques which allow small high-speed objects to be detected in close proximity to large and slow-moving objects. It organizes the measured samples into an m by n matrix. Figure 2 is an illustration of such a matrix. The m by n data cubes in the time domain is shown in the top half of the diagram. Each data sample in the “range samples” axis represents the distance from the radar antenna.

When the radar illuminates a target, multiple returns are received and processed. The “pulse interval” axis represents the range to the target extracted from the returning radar pulses. Each column in the “pulse interval” axis represents the individual samples taken between each transmit pulse [18,19,20]. There is an individual cube for each pulse repetition interval (PR). Since the Pulse-Doppler radar measures the Doppler shift and the fact that the target is moving radially, there will be a time shift between each pulse, which results in a phase shift over multiple returns. The time samples are converted to the frequency domain using a digital processor. This involves the Fast Fourier Transform (FFT) algorithm.

## 3. Experimental Results

### 3.1. Remote Vibrations Monitoring Using Doppler Technique

The vibration of the vehicle was detected using the speckle method with respect to the conventional Doppler method at a range of 80 m. Figure 2 presents the Doppler result of each range gate. To calibrate the radar for the conventional Doppler method, as well as for the speckle method, the moving target is located next to the static target in order to identify the location (i.e.*,* the Doppler range) of the static target.

Figure 2 also shows the Doppler frequency at a range of 80 m. The maximum response at 80 m was due to the DC response. It was monitored at all ranges, to see if the target location can be detected with the Doppler method. Please note that the car and person are located at 80 m from the radar. The *Y* axis of Figure 2 presents range Doppler cells. Each range cell represents 40 m. Hence, the target and the person are shown in range doppler cell 2.

It can be seen that when the conventional Doppler method is used, only a moving target is detectable while a static target is unseen.

**Figure 3:**
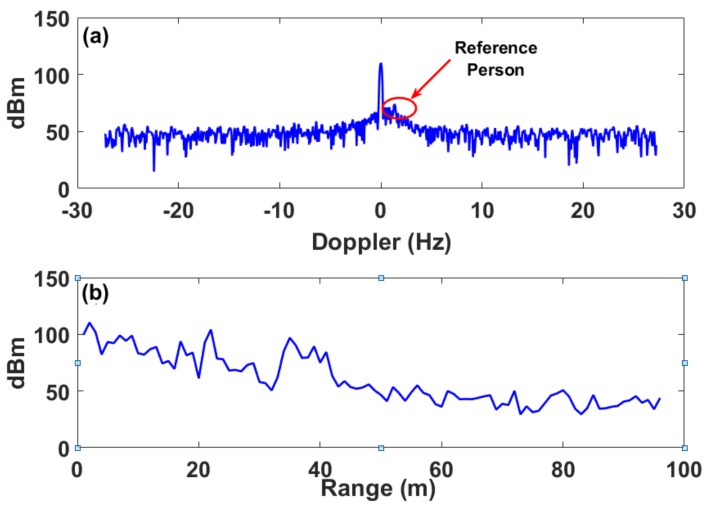
(**a**) Section along the Doppler frequency axis at the vehicle location when the engine is off. Note that the maximum response is shown as an unsymmetrical peak value adjacent the 110 dBm peak value, and relates to the moving reference person (red circle). (**b**) Section along the Doppler range axis at 0.01 Hz.

As can be seen in Figure 3, only the moving person was detected (an unsymmetrical high peak value near the 110 dBm), while the static vehicle was not detected. The Doppler frequency of the calibrated person measured in our experiments was approximately 3 Hz. The reference person walked relatively fast, hence, the Doppler signal in this case was clear.

**Figure 4:**
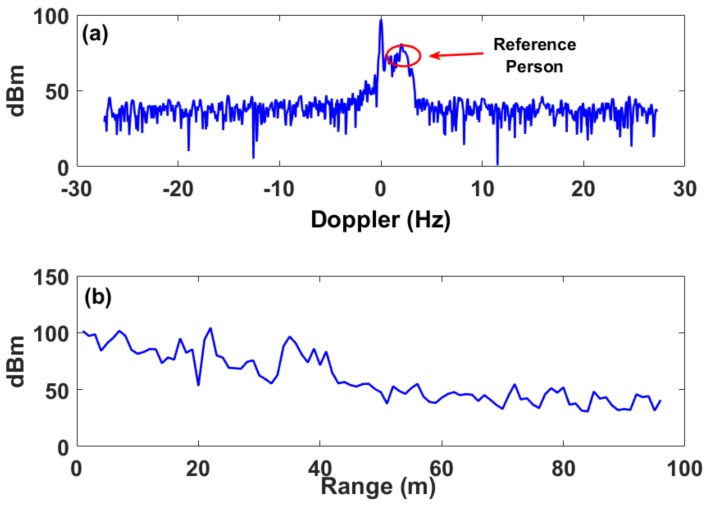
(**a**) Section along the Doppler frequency axis at the vehicle location when the engine is on. Note that the maximum response is shown as an unsymmetrical peak value adjacent the 110 dBm peak value and relates to the moving reference person (red circle). (**b**) Section along the Doppler range axis at 0.01 Hz.

The same process was repeated while the vehicle engine was on. The Doppler response at the target location (i.e., 80 m) was analyzed showing the highest response at 0.01 Hz as seen in Figure 3a. However, this response is not visible in the current Doppler method, and cannot be extracted as shown in Figure 4a.

Using the conventional Doppler method, when the vehicle engine was on, the target vibrations were not detected. However, when our **“**RF speckle**”** method is used, one is able to detect the vibrations in both cases.

### 3.2. Remote Vibrations Monitoring Using RF Speckle

As mentioned earlier, the temporal movement of the reflecting surface causes temporal changes in the random speckle pattern. The algorithm consists of the following steps: (a) Extracting the raw data of the specific range. The raw data consists of eight temporal radiating elements. (b) Calculating the 1-D correlation vector between two adjacent elements. (c) Extracting the temporal peak location from the correlation results. (d) Calculating the frequency response of the peak location. This process was repeated for each range value (i.e., 40, 80, and 120 m from the radar). 

**Figure 5:**
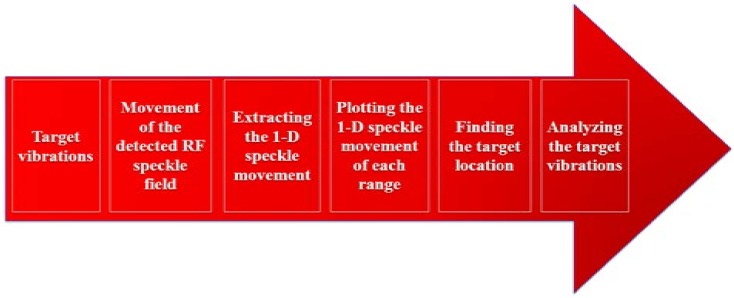
Summary of the speckle processing algorithm.

Initially, a temporal set of signals was transmitted by an array of eight different antennas. Please note that only the RF speckle raw data was extracted from the radar in the following technique. The array was used to receive the reflected temporal signals from the vibrated target simultaneously. Summary of the proposed technique is shown in Figure 5.

**Figure 6:**
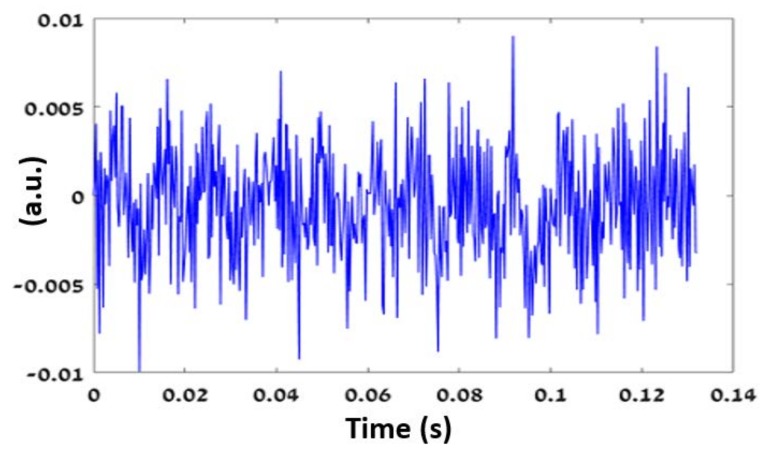
Temporal vibrations of the target with engine ignition off. The y-axis represents the arbitrary units of the temporal RF speckle pattern movement.

Each measurement contained 500 different temporal signal samples (i.e., each sample contained a 1-D vector of 8 bits, while each bit represented a different antenna). Later, by calculating the correlation, the relative movement of the target was extracted. This relative movement is obtained by allocating the time varying position of the 1-D correlation peak. The temporal movement of the target is proportional to the relative shift of the speckle pattern [1]:

(1) $$\beta \;=\frac{4\pi \tan\alpha }{\lambda }\;\approx \;\frac{4\pi \;\alpha \;}{\lambda }\;$$

where *β* is the change in the speckle pattern, *α* is the tilting angle of the illuminated surface and *λ* is the illuminating signal. The tilting movement presents additional linear phase. To sense the tilting movement, the signal was measured in the far field, and the linear phase was transformed to movement in the *X*–*Y* plane. Therefore, tracking the movement of the detected pattern is an indication for the tilting movement of the target due to small vibrations. The temporal movement of the target when the engine was turned off is shown in Figure 6. 

**Figure 7:**
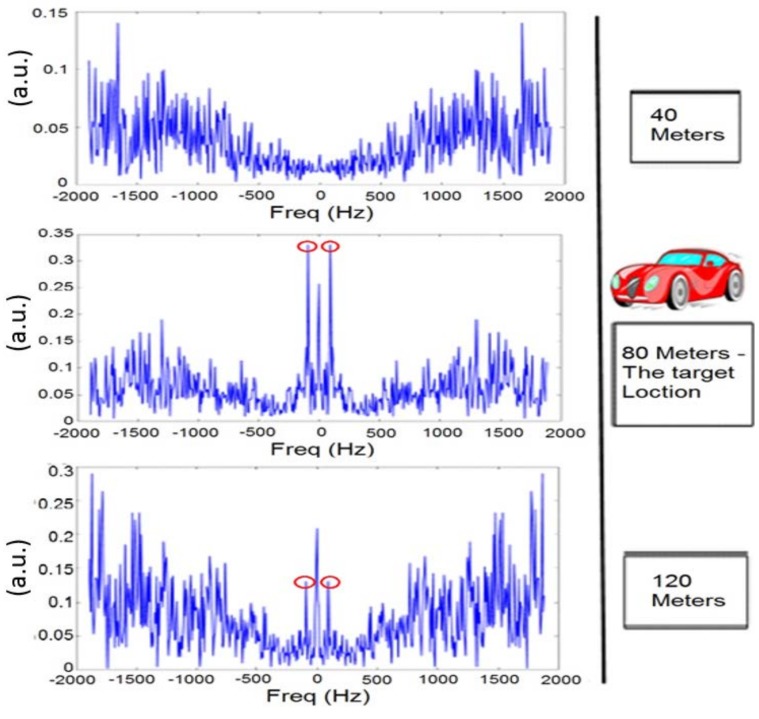
Frequency response at three different locations with engine ignition off.

Initially, the target was located at 80 m from the radar and the engine was turned off. One can see in Figure 7 the frequency response at three different locations: 40, 80 (the target location), and 120 m. A clear peak is shown only at the 80 m location (as indicated by the red circles), while the signals of the other locations show a noisy signal with very low SNR (signal-to-noise ratio). 

**Figure 8:**
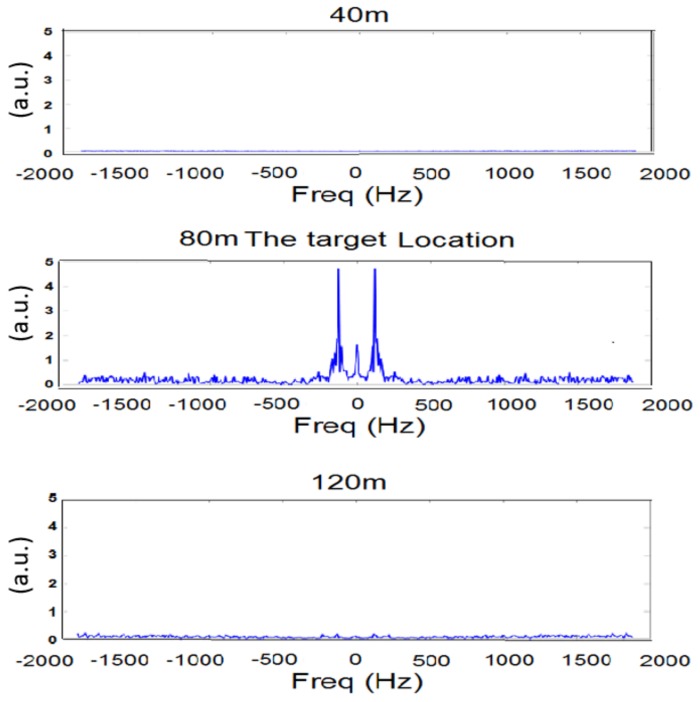
Frequency response at three different locations with the engine ignition turned on.

During the second step, the engine was turned on. The frequency response as a function of the distance from the radar is shown in Figure 8. 

One can see a maximum peak at 90 Hz only when the signal is received from the target (80 m from the radar). Furthermore, it can be shown that this peak is significantly higher than the peak measured when the engine was turned off. Figure 7 represents the frequency response of the temporal RF speckle movement. This calculation does not calculate the doppler effect. The result of Figure 7 shows that also when the car engine ignition was off, the vibration of the car due to the wind was detected. Please note that this movement was significantly smaller with respect to the case when the engine ignition was on. In Figure 7, we can sense small vibrations at 120 m. These vibrations are due to the fence vibrations at about 120 m from the radar using the speckle method. In Figure 8, one can see that the fence vibrations are negligible with respect to the vibrations of the target when the engine ignition was on. 

**Figure 9:**
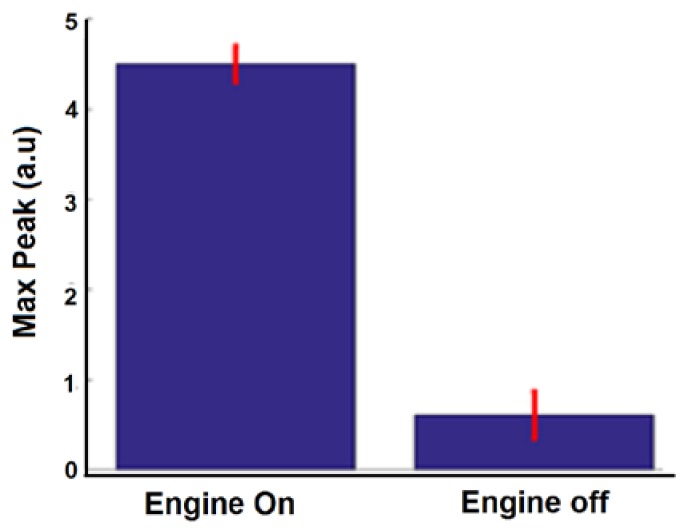
Difference between the maximum peaks when the car’s ignition was turned on and off.

This experiment was repeated five times (each time the car was turned on and off). The difference between the maximum peaks while the car was turned on and off is shown in Figure 9. 

## 4. Discussion and Conclusions

As mentioned earlier, the most common method for extracting and separating remote vibrations is to use the optical approach. In this experiment, we have demonstrated the ability to extract and separate remote vibrations by using RF speckles. The experiment consisted of illuminating a vehicle with Pulse Doppler radar in two different scenarios. One scenario with the engine turned on, and the other scenario with the engine turned off. Using an algorithm which analyzes the temporal changes of the random speckle patterns, we were able to extract the vibration frequency of the vehicle at a distance of 80 m. This experiment demonstrates the possibility of utilizing RF sources to detect and extract remote vibrations frequencies at far greater distances than were previously accomplished. In future work, we aim to use RF sources at higher frequencies than used in our experiment (>10 GHz). This will increase the resolution and sensitivity of the method and improve the results.
